# Calving Ease Risk Factors and Subsequent Survival, Fertility and Milk Production in Italian Holstein Cows

**DOI:** 10.3390/ani12060671

**Published:** 2022-03-08

**Authors:** Monica Probo, Marcello Guadagnini, Giulia Sala, Paola Amodeo, Agostino Bolli

**Affiliations:** 1 Department of Veterinary Medicine and Animal Science, University of Milan, Via dell’Università 6, 26900 Lodi, Italy; giuliasala.vet@gmail.com; 2 Elanco Animal Health, Via dei Colatori, 12, 50019 Sesto Fiorentino, Italy; marcello.guadagnini@elancoah.com; 3 Independent Researcher, Via Carpaccio 3, 20133 Milan, Italy; paamodeo@gmail.com; 4 Alta Italia s.r.l., Via Mascherpa 10, 20067 Paullo, Italy; agostino.bolli@altagenetics.com

**Keywords:** calving ease, fertility, Italian Holstein cows, milk production, risk factors, survival

## Abstract

**Simple Summary:**

Several studies have analyzed the potential risk factors for assisted calvings and the consequences of calving ease on cow performances. The present study used national data to focus on Italian Holstein cow herds. In summary, herd, number of calvings/herd/year, cow parity, gestation length, twinning, calf sex, previous calving-to-conception interval, dry period and close-up length, and calving season were identified as risk factors associated with calving ease. Regarding lactation performance, cows with assisted calving had higher 30 days in milk (DIM) culling risk, lower 150 DIM pregnancy risk and lower milk yield, measured as 60-d cumulative and as 305-d predicted milk yield. These results may be beneficial to focus attention on control, management and specific factors associated with calving ease in Italian Holstein herds and also to support adequate training and education of the personnel.

**Abstract:**

The objectives of this study were to investigate the main risk factors associated with calving ease (CE) in Italian Holstein cow herds, and to estimate the association between CE and subsequent survival, fertility and milk production. Data obtained from Holstein cows in 40 Italian herds were retrospectively investigated. Calvings were surveilled and classified into two categories of CE, unassisted calving or assisted calving, based on the need for intervention. The following factors were analyzed as possible risk factor affecting CE: herd, number of calvings/herd/year, age at first calving (AFC), cow parity, gestation length, twinning, calf sex, previous calving-to-conception interval, previous milk yield, dry period and close-up length, and season of calving. The association between CE and culling risk within the first 30 days-in-milk (DIM), cumulative 60-d milk yield and predicted 305-d milk yield, and pregnancy risk within 150 DIM were also investigated. Of the 47,672 calvings, 37,892 (79.5%) were unassisted, while 9780 (20.5%) required some type of assistance. Among the risk factors, only the AFC was not correlated with CE, while for all the other risk factors an association with CE was detected. Assisted calvings were associated with an increased culling risk at 30 DIM, decreased 60-d milk yield, decreased 305-d milk yield and reduced pregnancy risk at 150 DIM. In conclusion, dairy herd management should aim at correcting/reducing the risk factors in order to limit the incidence of assisted calving, and possibly improve the quality of calving assistance; controlling CE within the herd is crucial to reducing culling risk, and achieving higher lactation and reproductive performance.

## 1. Introduction

Genetic improvement of livestock during the second half of the 20th century using pedigree and performance data has been very successful, particularly in dairy cattle populations [1]. The primary selection emphasis for dairy cattle is on maximizing genetic gain and income through yield traits, but economic efficiency can be further enhanced by adequate emphasis on secondary traits that directly or indirectly affect production. Calving ease (CE) is one of the most economically significant secondary traits that directly influences the profitability of herds and the animals’ welfare [2,3]. Its estimated heritability ranges from 0.03 to 0.20 [4,5,6], and it has no genetic correlation with yield traits [6]. The economic importance of CE in the dairy industry concerns not only the short-term farm profits through the loss of calf, death of dam, veterinary fees, and extra labors [7,8], but also the long-term animal performance (i.e., health issues, fertility problems, reduced production, premature culling) [9].

At the herd level, control of dystocia is dependent upon specific sire selection, heifer growth and dry period management [10]. Moreover, farmers’ education for adequate periparturient management and obstetrical assistance is mandatory [11]. Veterinary literature is rich in investigations regarding risk factors and consequences of dystocia in dairy and beef cattle, but often different CE scores and dystocia definitions are employed, hampering comparisons. Dystocia scoring systems can in fact vary from two-point [12] to seven-point [13] scales, with thresholds of two or greater generally considered as assisted calving, and three or greater considered as a difficult calving. What is evident from the general research is that any calving assistance can be associated with reduced subsequent fertility and productivity [14]. Milk yield losses after a difficult calving have been quantified previously [15,16,17]; however, estimates are generally referred only to cases of dystocia, thus underestimating the actual need for calving assistance.

A recent investigation reported prevalence and risk factors of dystocia in Italian herds [18], but different cattle breeds were enrolled, and lactation performances were not evaluated. The hypothesis was that, by analyzing data obtained from a unique cattle breed and registered through a widespread herd management software system, it would be possible to better describe CE in Italian dairy herds. The objective of this work was therefore to retrospectively investigate the CE in Italian Holstein cows, to assess its correlation with specific risk factors and to estimate the effect of CE on subsequent survival, fertility and milk production.

## 2. Materials and Methods

### 2.1. Herds and Cows Data

This retrospective observational study involved the analysis of culling, fertility and milk production data collected from January 2019 to December 2020. Recruitment criteria for dairy herds were: a minimum size of 100 cows; the use of Dairy Comp 305^®^ (Valley Ag Software, Tulare, CA, USA) to record herd data for a minimum of 2 years previously; membership of the Italian National Breeders Association for milk production (AIA). According to these criteria and on the basis of their consistency over time in recording CE, a total number of 40 free-stall dairy herds spread throughout Italy were selected out of 63 originally participating in the project; in order to standardize the data, only records referring to Holstein–Friesian cows were considered and lactations with less than 260 days of gestation length were excluded.

In all herds, except 3, cows were fed a corn silage and haylage based diet with forage percentage ranging from 40% to 55% (as fed). In the other 3 herds, as their milk production is directed to Parmigiano Reggiano production, cows were fed a dry diet based on alfalfa hay, grass hay and concentrates with forage percentage ranging from 33% to 45%, according to the Italian product rulebook that does not allow the use of any ensiled ingredients.

For each lactation, the following data were considered: CE (see Section 2.2), herd, number of calvings/herd/year (categorized in <500 calvings/herd/year, 500–1000 calvings/herd/year, >1000 calvings/herd/year), age at first calving (AFC), cow parity (1, 2, ≥3), calving season (categorized as winter, spring, summer and autumn according to the northern hemisphere), gestation length (categorized as <274 d, 274–279 d, >279 d), twinning, calf sex in singleton calvings, previous lactation days open (previous calving-to-conception interval categorized as ≤200 d and >200 d), previous 305-d mature equivalent (ME) milk yield, dry period length (categorized as <44 d, 44–70 d, >70 d) and close-up (categorized as <18 d, 18–25 d, >25 d) length, culling risk within the first 30 days-in-milk (DIM), 60-d cumulative milk production and predicted 305-d milk yield, and pregnancy risk within 150 DIM. For each, continuous variable classes were established based on data distribution and usefulness of the categorization.

### 2.2. Calving Ease

Farmers or specific technicians, upon observing parturition, assigned a CE score according to the degree of assistance provided. Calvings were first classified according to the National Breeders Association standards (Associazione Italiana Allevatori, AIA, Roma, Italy) in unassisted calvings, calvings needing manual intervention, calvings needing a caesarean section, and calvings needing a fetotomy. Subsequently, in order to avoid scatter of data, calvings were merged into two groups, namely calvings needing for intervention, or assisted calvings, and unassisted calvings. As stated by [10] assisted calving may be defined as a calving where assistance is rendered, although this may not result in dystocia.

### 2.3. Fertility and Milk Production

After a voluntary waiting period specific for each herd, cows were submitted to different protocols for the first insemination using frozen semen. In 4 of the enrolled herds, cows were submitted to a Presynch-OvSynch protocol [19,20], while in 25 herds cows were subjected to a Double-OvSynch protocol [21]. In 2 herds, insemination was performed using a combination of estrus detection and Ovsynch protocol, while in 5 herds cows were inseminated using only estrus detection. In 4 herds the reproductive management was changed over time during the observation period from estrus detection only to timed AI using Double-Ovsynch. The second and further inseminations were performed on estrus detection using sensors and/or Ovsynch. Heifers were inseminated using female selected sex-sorted semen in all herds, except four. 

Daily milk was monitored through milk meters. Monthly milk recording was performed by trained technicians of the local milk test day organizations, and according to the guidelines of the International Committee for Animal Recording (ICAR) [22].

### 2.4. Statistical Analysis

Data regarding each single herd were exported from Dairy Comp 305^®^ herd management software to a Microsoft Excel^®^ file (Microsoft Corp., Redmond, WA, USA), and all statistical analyses were performed using JMP15.1^®^ (SAS Institute Inc., Cary, NC, USA). Descriptive statistics were performed and continuous variables were expressed as the mean ± standard deviation (SD), while categorical variables were expressed as frequencies and percentages. Statistical difference between herds was assessed through χ2 test. Studied risk factors for assisted calvings were: number of calvings/herd/year, AFC in months (only for primiparous cows), cow parity, calving season, gestation length, twinning, calf sex in singleton calvings, previous lactation days open (previous calving-to-conception interval), dry period and close-up length. For each risk factor, a dedicated multiple logistic regression model has been made in order to address its correlation with CE, accounting for specific other explanatory variables. The statistical model used for each risk factor and the variables included in each model are reported in Table 1. Moreover, using dedicated multiple linear regression models, the correlation between milk production in the subsequent lactation and other variables, including CE, has been studied (Table 2). The association between CE and time to conception was censored at 150 DIM, using a separate Cox proportional hazard model for primiparous and multiparous cows. Likewise, the correlation between CE and time to culling within 30 DIM was described using a Cox proportion Hazard model for first lactation (LACT) animals and a different one for multiparous cows. The statistical approach used to correlate CE with reproduction, culling and the variables included in each model are reported in Table 2. The significance and tendency levels were set at *p* < 0.05 and *p* < 0.1, respectively.

## 3. Results

### 3.1. Descriptive Statistics

The minimum size of the enrolled herds was 121 cows, the maximum was 1921 cows (mean ± SD: 690.45 ± 151.32). Ultimately, the data set included a total of 47,672 calvings in a 2 year period, with 22,288 calvings taking place in 2019 and 25,384 occurring in 2020. The mean number of lactations analyzed for a single herd was 1192 ± 680, ranging from 430 to 3865 lactations. The mean (±SD) 305-d milk production of the herds was 10,782 (±2178) kg/cow. Assisted calvings accounted for a total of 9780 (20.5%), while 37,892 (79.5%) did not require assistance. 

### 3.2. Risk Factors for CE

**Figure 1:**
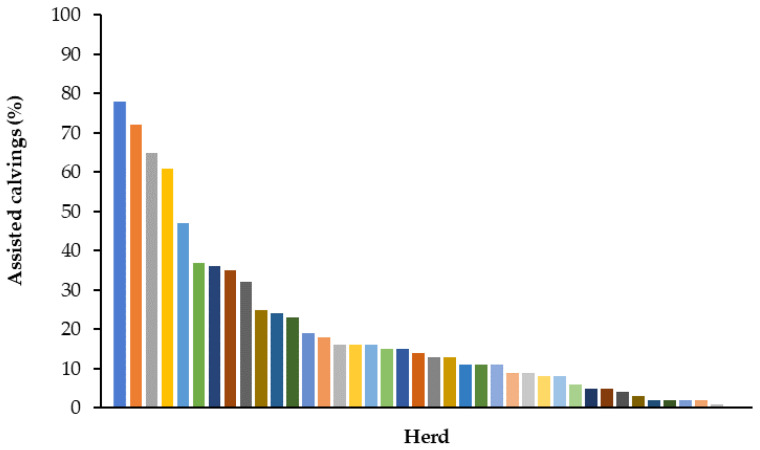
Distribution of assisted calving prevalence in the 40 Italian Holstein cow herds enrolled in the study. Each column corresponds to a single herd.

Distribution of assisted calving prevalence among the 40 herds is reported in Figure 1. The odds ratios (OR) and confidence interval (95% C.I.) for the association between evaluated factors and CE are reported in Table 3. Calving ease was associated with herd factor (*p* < 0.0001), and with the number of calvings/herd/year categories (*p* < 0.0001). Mean (±SD) AFC, referred to the 17,159 primiparous cows, was 24 (±2.11) months; 52% (*n* = 8938) of heifers calved at an age between 23 and 26 months, 33% (*n* = 5705) calved at less than 23 months, while AFC was >26 months in 15% (*n* = 2516) of the heifers. According to the statistical analysis, AFC in categories was not associated with CE score (*p* = 0.283). A total of 36% (*n* = 17,159) of the lactation records belonged to primiparous cows (LACT = 1), 27% (*n* = 12,770) to second parity cows (LACT = 2), and 37% (*n* = 17,743) to ≥third parity cows (LACT = 3+). Calving ease was associated with parity, as assisted calving prevalence increased with increasing parity (*p* < 0.0001). The OR (95% C.I.) for assisted calving in LACT = 3+ cows was 1.24 (1.16–1.32) when compared to LACT = 2 cows, and 1.29 (1.21–1.37) when compared to LACT = 1. The OR remained unvaried also forcing models by introducing calf gender as a factor. No differences were found in CE between first and second parity cows (*p* = 0.263). Mean (±SD) gestation length was 275 ± 9 d; cows with a gestation length >279 d (*n* = 12,719; 27%) had greater risk of assisted calvings compared to cows with a gestation length ranging from 274–279 d (*n* = 21144; 44%) and to cows with a gestation <274 d (*n* = 13,809; 29%). In 97.3% of cases, calvings were singleton, and gave birth to 26,109 female calves (56.4%) and to 20,196 (43.6%) male calves. Calf gender in singleton calvings was associated with CE, as male calves were more likely to require assistance at birth than female calves (OR (95% C.I.): 1.39 (1.30–1.48)). A total of 82% of female calves were delivered without assistance compared to 78% of male calves. Twinning occurred in 0.53% of LACT = 1 calvings, in 2.79% of LACT = 2 calvings and in 4.61% LACT = 3 + calvings. Twin calvings (*n* = 1262; 2.7%) were more likely to require assistance than singleton (OR (95% C.I.): 3.71 (3.21–4.28)). The risk of assisted calvings was higher in cows with >200 previous lactation days open (89% of the total) compared to cows with ≤200 previous lactation days open (11% of the total) (OR (95% C.I.): 1.33 (1.21–1.47)). Cows calving in autumn had lower risk for assisted calving compared to those calving during winter (*p* < 0.01) and spring (*p* < 0.01), and with a tendency to a lower risk when compared to the summer season (*p* = 0.0505). 

Dry period length was correlated with CE, as cows with a dry period >70 d had higher risk of assisted calving compared to cows with a 44–70 d dry period (OR (95% C.I.): 1.26 (1.15–1.38)). A long (>28 d) close-up period was associated with greater risk for assistance at calving compared to short (<15 d) close-up period; *p* < 0.05) or with 15–28 d close-up period (*p* < 0.01). 

### 3.3. Associations between CE and Survival, Fertility and Milk Production

Multiparous cows with assisted calving had a higher risk of being culled in the first 30 days after calving; the risk was even higher when cows were primiparous. Pregnancy risk within the first 150 DIM was reduced in cows with assistance at calving compared to those not requiring assistance. CE was also associated with 60-d cumulative milk yield and estimated 305-d milk yield both in primiparous and multiparous cows. Data on risk ratios for the association between CE and survival and fertility in primiparous and multiparous cows are reported in Table 4. Estimated least squares mean regarding the associations between CE and milk production in primiparous and multiparous cows are reported in Table 5.

## 4. Discussion

The overall prevalence of assisted calvings in this study was 20.5%, much higher than other data reported on Italian herds [2,18]. Cow breed/attitude and definition criteria for assisted calvings are key factors to consider. The study by Carnier et al. [2] was focused on Piedmontese cows; breeds and attitude are recognized as influencing factor for dystocia, so that when attitude is considered, the prevalence of dystocia is higher in dairy cows than in beef cattle [18]. Inclusion criteria, definition, and dystocia scoring vary greatly among studies, and may be partially responsible for the different prevalence of assisted calvings found in the present study compared to previous studies in Holstein cows [17,18,23]. De Amicis et al. [18] reported a dystocia prevalence of 6.2% in Holstein cows, but only true cases of dystocia were included. In the study by Atashi et al. [17], calvings presenting slight problems were classified as unassisted calvings, while in the present study all calvings requiring human intervention, albeit slight, were considered as assisted. This grading method aimed to avoid the scattering of data; moreover, it also allowed the registering of the prevalence of calvings that needed any type of assistance, although not necessarily resulting in dystocia, and it is therefore, to the best of our knowledge, the first report of CE in the Italian Holstein breed. A higher prevalence of assisted calvings was recorded by other authors in population studies in which Holstein Friesian represented the vast majority (>95%) [24] or the only breed included [25].

At the herd level, dystocia rates usually follow a positively skewed distribution [10] with a substantial proportion of herds having a low prevalence and a small proportion of herds with a high prevalence. The present data confirmed this distribution for CE (see Figure 1), with calving assistance herd prevalence ranging from 0.8% to 78.3%, underlying the importance of herd management in CE control. This is confirmed by the increasing prevalence of assisted calvings found with the increasing number of calvings/herd/year; it may be assumed that timeliness and procedures implemented by the herd technical staff in calvings surveillance differ from smaller to larger herds. Therefore, specific training of technical staff by veterinarians and animal specialists would be desirable in order to improve calving management.

The AFC did not affect CE in the present study, and this is in contrast with previous studies. Atashi et al. [26] found that, compared to heifers calving at 23 to 26 months of age, both increasing and decreasing AFC were associated with increased risk of dystocia. Again, differences in the criteria used for definition of AFC classes may be the reason for different results. Moreover, gestation length in heifers is curvilinear [27], giving rise to a shorter gestation in both young (20.3 < AFC < 22 months) and old animals (AFC ≥ 25.5 months) compared with the intermediate-age group (22 < AFC < 25.5 months), which further contributes to the lower birth weight of calves born to relatively young and old first-parity dams. The incidence of dystocia is also affected by age in a curvilinear manner, with young and older heifers being more affected [28,29]; higher calving difficulties in very young and older heifers are possibly due to immaturity of the young dams or to the excessive fat deposition in the pelvis of old heifers, respectively [30]. In the present study, a possible AFC effect on CE might therefore be reversed by the use of female sex-sorted semen in almost all heifers, which gave birth to higher number of females; since female calves are lighter than male calves at birth [31,32,33], the importance of AFC within CE evaluation in the present study may have been mitigated. Moreover, growth rate and body development in heifers is not only correlated with age, but also with genetics, nutrition and health [34].

A parity effect on CE was present, but in disagreement with previous studies, as we found higher risk of assisted calvings in LACT = 2 and LACT = 3+ cows compared to LACT = 1, with LACT = 3+ cows accounting for the majority of calving difficulties. Associations between parity and calving difficulties in the literature is reported, instead, as a decreasing percentage of dystocia with an increasing parity of the cow [24,35,36], and with the highest probability of difficult calving in first parity cows [36]. A possible explanation may lie within the concept that calf gender and dam parity interact with CE affecting stillbirth [24]. Thompson et al. [37] in fact, reported a different association between calf size and stillbirths for primiparous and multiparous cows. According to these authors, large calves had greater mortality when delivered by first parity cows, and small calves had greater mortality when delivered by cows with parities greater than 1; size of the birth canal is less restrictive in later parities, thus giving preference to larger, better-developed calves, which would be at greater risk in primiparous cows. In most of the herds enrolled in the present study, heifers were inseminated using mostly female selected sex-sorted semen, as it can be inferred from the female/male ratio in calves born from primiparous cows (70.4% females vs 29.6% males). When exploring the association between calf sex and CE in the present study, cows calving bull calves were found to be at greater risk for assisted calving, in agreement with previous literature [23,29,38]. Consequently, the high percentage of female calves in primiparous dams possibly influenced CE, reducing the need for assistance at calving, especially in comparison with multiparous cows in which sex ratios of calves were balanced (51% female calves in LACT = 2) or with more males (46% female calves in LACT = 3+). Also, Meyer et al. [35] found that when assistance was needed at birth, the dystocia affected the calves of multiparous cows more than the calves of primiparous cows. Finally, across Italian herds it is a common practice to inseminate older cows using beef bull semen, which could deliver larger calves compared to dairy bull semen. Unfortunately, this information was not available for the present study; although a weak correlation between sire breeding value for CE and birth-weight of the calves was found [27], it was suggested that the uterine environment has a greater influence on size at birth than the paternal genotype [27,39]. 

The twinning rate detected in the present study (2.7%) was similar to those previously reported [40] (2.5%), [13] (3.11%); twins were more likely to require assistance during calving than singletons, which is in agreement with many studies [13,16,23,38,41,42]. The majority of calving difficulties with twins results from abnormal presentation of one or both fetuses at delivery rather than from large birth weight or physical size [41]. Reducing the untoward consequences of twinning can be achieved by ovulation synchronization protocols [43], periparturient diagnosis of twin pregnancies, (which allows timely administration of obstetrical assistance), and by genetic selection (avoiding selection of dams with multiple ovulations). 

Gestation length categories were associated with CE: cows having gestation length >279 days had an increased risk of assisted calvings when compared to gestations ranging 274–279 days. Possibly, this is related to the fact that fetal body weight increases exponentially with gestational age in cattle [44,45], with an average fetal daily weight gain of 0.5 kg in the last week of gestation [32,46]; calves born before term are often smaller than calves at term, and in those cases, calving is also unexpected, so most likely not assisted. Many other factors such as season and genetics may cause variation in gestation length, and fetal oversize may be hypothesized to be a risk factor for those gestations exceeding 279 d. Very short (<260 d) gestations were not considered, thus eliminating those cases in which calving assistance was caused by abortion and fetal mortality, as bovine abortion is defined as the expulsion of a fetus between the completion of differentiation (day 42) and the limit of fetal independent viability (day 260) [46]. Although the risk for assisted calvings in the present study was reduced in short gestations (<274 d) when compared to a gestation length of 274–279 d, calf viability and welfare should also be considered; both short and long gestation have been associated with greater incidence of dystocia and stillbirth compared to average gestation length, due to reduced viability of the newborn or increased calf body weight, respectively [29,35,47]. Intermediate gestation length, namely between 274 and 281 d, optimizes lifetime productivity, CE, incidence of stillbirth and the interval from calving to first service in dams [48]. For the purposes of this study, no investigation on the outcome of the calving was performed, as calving assistance was provided in different ways among the different herds. Many variables that could not be measured in this study could influence the outcome of assisted calvings, including magnitude of traction applied and timing of intervention. 

As many others, the present study reported a seasonal effect on CE in Holstein dairy cattle. In some investigations, a decreasing risk of dystocia was found in summer compared with colder seasons [29,49]. Cold weather during the last trimester has been associated with increased dry matter and energy intake, increased thyroid hormone concentration, increased blood and nutrient flow to the uterus and increased gestation length and reduced plasma estradiol concentrations, leading to increased birthweight and dystocia [29,50,51]. Consistently, we found a higher risk for calving assistance during winter and spring compared to autumn, similar to [13]. 

The higher risk for assisted calvings in cows with long dry periods (>70 d) supports previous findings [52], and it is probably due to the relationship between dry period length and cow body condition score (BCS) at calving. Longer dry and close-up periods lead to a BCS rise, with a body fat deposition along the birth canal that may increase calving difficulties.

This study presented an overall increased 30 DIM culling risk for cows with assisted calvings, in agreement with previous studies [38,53]. It is true that the timing of diagnosis or withdrawal periods for disease treatments can bias the moment of culling and influence survival analysis in early lactation [54]; nevertheless, cullings due to poor lactation performances or infertility generally occur later during lactation, while cullings in early lactation depend mostly upon calving problems, transition diseases or metabolic disorders. The general effect of CE on 30 DIM culling risk was more pronounced in primiparous cows (1.68 RR in primiparous cows compared to 1.37 RR in multiparous cows), and this is in agreement with a previous investigation [55], although the definition of CE scores differs.

Fertility was also correlated with CE in the present study, as suggested by others [5,15,28,37,56,57]. Although the definition of CE scores differ among studies in several measures of fertility—days open, calving interval, number of services per conception, and days to first service—an increase in units following a difficult calving is concluded throughout. Difficulties at calving reduced 150 DIM pregnancy risk in this study, consistent with the literature. Cows experiencing difficulty at parturition are more likely to suffer from postpartum diseases such as metritis, retained placenta and milk fever; these health and metabolic disturbances delay ovarian recovery and uterine involution, impairing subsequent fertility [58].

The negative effect of calving assistance on milk production in the present study was evidenced both by the effective measurement of cumulative 60-d milk yield and by the predicted 305-d production. As reported by Atashi et al. [26], the repeated measurements for monthly test-day milk samples better estimates the milk loss compared to a summary measure such as 305-d milk yield, also because the first is a “real” estimation and not predicted data. Moreover, the same authors [26] suggested that dystocia was associated with decreased 305-d lactation performance mostly in early lactation. Nonetheless, cumulative 60-d milk yield data, despite the bias due to the exclusion of cows culled before 60 DIM, might be a useful metric to define early lactation milk production. In the present study, both milk production metrics pointed out the negative effect of assisted calvings on lactation performance. These results indicated that, although restrained in quantity, losses in milk production associated with assisted calvings are prolonged over time, and they are therefore biologically important. Although a general negative impact of dystocia on lactation performances has been established in the literature, the reports on the relationship between calving difficulties and lactation performances at different parities and different stages of lactation are not consistent [5,16,57,59]. Once again, different definitions of CE score and different statistical methods used to estimate milk loss could be the main sources of these discrepancies.

## 5. Conclusions

The present study focuses on CE in Italian Holstein cow herds, and to our knowledge, it is the first study in this regard. This study contributes to identifying specific factors associated with CE, showing that calving difficulties are associated with worsening of both survival and fertility and milk production performances; moreover, it promotes attention on calving management at the herd level and on herd staff training and education.

## Figures and Tables

**Table 1:** Description of statistical model and variables included for each risk factor considered in the study.

Risk Factor	Model Outcome	Model Type	Model Variables	N
Farm	Calving ease (Assisted/Unassisted)	Contingency analysis	Farm	47,672
Number of calvings/herd/year categories	Calving ease (Assisted/Unassisted)	Multiple logistic regression model	Number of calvings/herd/year in categories; parity group; calving year; calving month	47,672
Parity group	Calving ease (Assisted/Unassisted)	Multiple logistic regression model	Farm; parity group; gestation length in days; calving year; calving month	47,672
Gestation length	Calving ease (Assisted/Unassisted)	Multiple logistic regression model	Farm; parity group; gestation length in days; calving year; calving month	47,672
Gestation length in categories	Calving ease (Assisted/Unassisted)	Multiple logistic regression model	Farm; parity group; gestation length categories; calving year; calving month	47,672
Age at first calving categories (primiparous cows)	Calving ease (Assisted/Unassisted)	Multiple logistic regression model	Farm; age at first calving categories; gestation length categories; calving year; calving month	17,159
Previous lactation days open categories	Calving ease (Assisted/Unassisted)	Multiple logistic regression model	Farm; parity group; gestation length in days; previous lactation days open categories; calving year; calving month	30,500
Days dry categories	Calving ease (Assisted/Unassisted)	Multiple logistic regression model	Farm; parity group; gestation length in days; days dry categories; calving year; calving month; days dry categoriesgestation length in days	30,442
Days in close-up categories	Calving ease (Assisted/Unassisted)	Multiple logistic regression model	Farm; parity group; gestation length in days; calves number; calving year; calving month	20,220
Calves number (twin/singleton)	Calving ease (Assisted/Unassisted)	Multiple Logistic regression model	Farm; parity group; gestation length in days; calves number(twin/singleton); calving year; calving month	47,567
Calf sex (singleton calvings)	Calving ease (Assisted/Unassisted)	Multiple logistic regression model	Farm; parity group; gestation length in days; calf sex (singleton calvings); calving year; calving month	46,305
Calving season	Calving ease (Assisted/Unassisted)	Multiple logistic regression model	Farm; parity group; gestation length in days; calving season	47,672

**Table 2:** Statistical models and variables included for the analysis of the association between calving ease and survival, fertility and milk production.

Model Outcome	Outcome Variable	Model Type	Model Variables	N
Culling risk in the first 30 DIM primiparous cows	Time to culling	Cox proportional hazard	Farm; gestation length in days; calving year; calving month; calving ease; Age at first calving in months	17,159
Culling risk within the first 30 DIM multiparous cows	Time to culling	Cox proportional hazard	Farm; parity group; gestation length in days; days dry categories; calving year; calving month; previous lactation 305ME milk production; calving ease	29,708
Pregnancy risk within the first 150 DIM primiparous cows	Time to pregnancy	Cox proportional hazard	Farm; gestation length in days; calving year; calving month; age at first calving in months; calving Ease	17,159
Pregnancy risk within the first 150 DIM multiparous cows	Time to pregnancy	Cox proportional hazard	Farm; parity group; gestation length in days; days dry categories; calving year; calving month; previous lactation 305ME milk production; previous Days open categories; calving ease	29,975
60 DIM cumulative milk production (Kg) primiparous cows	60 DIM cumulative milk production (Kg)	Multiple Linear regression	Farm; gestation length in days; calving year; calving month; age at first calving in months; calving Ease	14,475
60 DIM cumulative milk production (Kg) multiparous cows	60 DIM cumulative milk production (Kg)	Multiple Linear regression	Farm; parity group; gestation length in days; days dry categories; calving year; calving month; previous lactation 305ME milk production; calving Ease	24,211
Predicted 305-d milk production (Kg) primiparous cows	Projected 305-d milk production (Kg)	Multiple Linear regression	Farm; gestation length in days; days in milk, calving year; calving month; age at first calving in months; calving Ease	15,043
Predicted 305-d milk production (Kg) multiparous cows	Projected 305-d milk production (Kg)	Multiple Linear regression	Farm; parity group; gestation length in days; days in milk; days dry categories; calving year; calving month; previous lactation 305ME milk production; calving ease	24,211
Culling risk in the first 30 DIM primiparous cows	Time to culling	Cox proportional hazard	Farm; gestation length in days; calving year; calving month; calving ease; age at first calving in months	17,159
Culling risk within the first 30 DIM multiparous cows	Time to culling	Cox proportional hazard	Farm; parity group; gestation length in days; days dry categories; calving year; calving month; previous lactation 305ME milk production; calving ease	29,708
Pregnancy risk within the first 150 DIM primiparous cows	Time to pregnancy	Cox proportional hazard	Farm; gestation length in days; calving year; calving month; age at first calving in months; calving Ease	17,159

**Table 3:** Risk factor associations with calving ease.

Factor	Number ^1^	Odds Ratio (95% C.I.) ^2^	*p*-Value
**Calvings/herd/year**	47,672		
<500	9815	Referent	<0.0001
500–1000	20,566	1.11 (1.04–1.18)	
>1000	17,291	1.42 (1.34–1.52)	
**Gestation length**	47,672		
274–279 d	21,144	Referent	<0.0001
<274 d	13,809	0.97 (0.91–1.04)	
>279 d	12,719	1.22 (1.13–1.31)	
**Parity**	47,672		
1	17,159	Referent	<0.0001
2	12,770	1.04 (0.97–1.11)	
3+	17,743	1.29 (1.21–1.37)	
**Previous lactation days open**	30,500		
≤200 d	27,036	Referent	<0.0001
>200 d	3464	1.33 (1.21–1.47)	
**Calf number**	47,567		
Singleton	46,305	Referent	<0.0001
Twins	1262	3.71 (3.21–4.28)	
**Calf sex (singleton calvings)**	46,305		
Female	26,109	Referent	<0.0001
Male	20,196	1.39 (1.30–1.48)	
**Calving season**	47,672		
Autumn	14,216	Referent	<0.0001
Summer	12,930	1.07 (1.00–1.15)	
Winter	10,806	1.12 (1.05–1.21)	
Spring	9720	1.13 (1.05–1.21)	
**Dry period length**	30,442		
44–70 d	24,787	Referent	<0.0001
<44 d	1109	1.03 (0.85–1.25)	
>70 d	4546	1.26 (1.15–1.38)	
**Close-up length**	20,220		
15–28 d	12,136	Referent	<0.0001
<15 d	5186	0.96 (0.85–1.07)	
>28 d	2898	1.22 (1.08–1.38)	

^1^ Number = number of observations in the dataset; ^2^ 95% CI: 95% confidence intervals for estimated value. Specific risk factors are reported with bold type in the table.

**Table 4:** Risk ratios and 95% confidence interval for the associations between calving ease and survival and between calving ease and fertility.

Parameter	Unassisted Calving	Assisted Calving	CI	Significance
30 DIM Culling Risk				
*Primiparous cows*	N = 13824	N = 3335		
	Referrent	RR 1.68	1.31–2.14	*p* < 0.0001
*Multiparous cows*	N = 23345	N = 6363		
	Referrent	RR 1.37	1.21–1.54	*p* < 0.0001
150 DIM Pregnancy risk				
*Primiparous cows*	N = 13824	N = 3335		
	Referrent	RR 0.94	0.88–0.99	*p* = 0.03
*Multiparous cows*	N = 23588	N = 6377		
	Referrent	RR 0.91	0.86–0.96	*p* = 0.0004

RR: risk ratios; CI: 95% confidence intervals for estimated value; parity groups are reported in Italics.

**Table 5:** Least square means and 95% confidence intervals for milk production according to calving ease.

Parameter	Unassisted Calving	CI	Assisted Calving	CI	Significance
Cumulative 60-d					
milk yield-LSM (Kg)					
*Primiparous cows*	N = 1854.3	1846.9–1861.6	N = 1833.8	1818.8–1848.7	*p* = 0.01
*Multiparous cows*	N = 2626.4	2619.6–2633.3	N = 2591.3	2577.9–2604.7	*p* < 0.0001
Predicted 305-d					
milk yield-LSM (Kg)					
*Primiparous cows*	N = 9633.5	9603.9–9663.0	N = 9557.7	9497.7–9617.8	*p* = 0.02
*Multiparous cows*	N = 11,297.6	11,427.6–11,476.1	N = 11,237.1	11,341.6–11,435.1	*p* = 0.02

LSM: least square mean; CI: 95% confidence intervals for estimated value; N = number of observations in the dataset; parity groups are reported in Italics.

## Data Availability

The data presented in this study are available upon reasonable request to the corresponding author.
